# Association between Bone-Related Physiological Substances and Oral Function in Community-Dwelling Older People

**DOI:** 10.3390/ijerph191710677

**Published:** 2022-08-27

**Authors:** Misa Nakamura, Masakazu Imaoka, Fumie Tazaki, Hidetoshi Nakao, Mitsumasa Hida, Ryohei Kono, Hideki Kanemoto, Masatoshi Takeda

**Affiliations:** 1 Cognitive Reserve Research Center, Osaka Kawasaki Rehabilitation University, Kaizuka 597-0104, Osaka, Japan; 2 Department of Rehabilitation, Osaka Kawasaki Rehabilitation University, Kaizuka 597-0104, Osaka, Japan; 3 Department of Physical Therapy, Josai International University, Tougane 283-0002, Chiba, Japan; 4 Department of Psychiatry, Graduate School of Medicine, Osaka University, Suita 565-0871, Osaka, Japan

**Keywords:** IGF-1, mastication, community-dwelling, older people

## Abstract

Background: Oral dysfunction is related to long-term cares including activities of daily living. The objective of this study was to determine the association between oral function and the bone-related physiological substances osteocalcin (OC) and insulin-like growth factor-1 (IGF-1). Methods: The study participants were 139 community-dwelling older people in Japan. Evaluation of oral dysfunction was based on subjective judgment by each participant. Blood analysis included OC, IGF-1, and albumin. Results: Univariate and multiple logistic analyses showed that IGF-1 was significantly associated with a “decline in masticatory function” (*p* = 0.0074 and *p* = 0.0308, respectively). Receiver operating characteristic curve analysis of IGF-1 levels revealed a threshold score of 108 ng/mL (*p* < 0.01) for discriminating a “decline in masticatory function”. Logistic regression analysis revealed that participants with an IGF-1 level ≤108 ng/mL had an odds ratio of 4.31 (*p* < 0.05) for a “decline in masticatory function”. No significant association was found between the OC level and oral dysfunction. Conclusions: These results suggest a possible relationship between lower serum IGF-1 levels and a decline in masticatory dysfunction in community-dwelling older people.

## 1. Introduction

The need for elderly care is a major problem facing societies around the world. Causes of the need for support and care include falls, fractures, and other disorders that affect exercise, whereas causes of motor dysfunction include age-related loss of muscle strength and balance and conditions such as osteoporosis, osteoarthritis, sarcopenia, and osteoporosis [1].

Bone remodeling is continuously performed through osteoblastic bone formation and osteoclastic bone resorption. Osteocalcin (OC) is secreted by bone osteoblasts and used as a biomarker of the osteogenic process [2]. On the other hand, insulin-like growth factor-1 (IGF-1) is produced in many tissues, including osteocytes and muscle cells, and it functions as a myokine to regulate muscle protein synthesis [3,4,5]. Therefore, muscle hypertrophy and bone formation are thought to be synchronized by the IGF-1 paracrine signal [6]. Recently, it has been reported that strong chewing in rodents increases the expression of IGF-1 in osteocytes [7].

The results of several cohort studies revealed relationships between masticatory dysfunction and poor oral condition, decreased tooth numbers and physical dysfunction, and tooth loss and mortality [8,9,10]. The World Health Organization recommends the development of interventions to improve oral health [11]. Furthermore, oral frailty, which was proposed by the Japanese Ministry of Health, Labor, and Welfare in 2013, is one aspect of frailty that include slight deterioration of oral function and change in food take [12]. Some studies have shown the relationships between oral health and bone mineral density [13], and osteoporosis [14], as well as malnutrition [15], sarcopenia [16], weakness [17], depression [18], and cognitive decline [19,20]. Serum IGF-1 levels are associated with sarcopenia [21], and frailty [22].

Therefore, with the goal of clarifying biomarkers of oral dysfunction in community-dwelling older people, the objective of this study was to determine the associations between oral function and the bone-related physiological substances OC and IGF-1.

## 2. Materials and Methods

### 2.1. Participants

The present study was conducted in Kaizuka city, Osaka Prefecture, Japan, between 2019 and 2020. The inclusion criteria were as follows: (1) age ≥ 60 years; (2) living independently at home; and (3) not having a cardiac pacemaker, which is affected with the method for measuring body composition. All participants participated in an annual health checkup co-sponsored by Osaka Kawasaki Rehabilitation University and the Kaizuka City Elderly Care Division. Of the 142 participants, 139 (mean age ± standard deviation, 74.16 ± 5.70 years; range, 61–91 years) were analyzed, after excluding three who had incomplete data. This study was approved by the Ethics Committee of Osaka Kawasaki Rehabilitation University (Reference No. OKRU30-A016) and performed in accordance with the Declaration of Helsinki. Written informed consent was obtained from all participants before the study began.

### 2.2. Measurement of Variables

Body composition parameters were measured using a bioelectrical impedance analysis (BIA) device (InBody 270; InBody, Tokyo, Japan) at 20 and 1000 kHz while the participants were wearing normal indoor clothing without socks or shoes. All participants were instructed to grasp the handles of the BIA device and stand on electrodes contacting the bottoms of their feet. Body mass index (BMI) was calculated as weight in kilograms divided by height in meters squared. The skeletal muscle mass index (SMI) was calculated as muscle mass in kilograms divided by height in meters squared. Calcaneus bone mineral density (BMD) was evaluated by quantitative ultrasound (i.e., the speed of sound [SOS] of the calcaneus) and expressed as the percent of the young adult mean of the SOS (%YAM) using an ultrasound bone densitometer (AOS-100SA; Hitachi, Tokyo, Japan).

### 2.3. Evaluation of Depression

Depression status was assessed using the Geriatric Depression Scale (GDS-15), which is a commonly used instrument for depression screening in the general geriatric population [23].

### 2.4. Blood Examination

Blood samples were drawn between 10:00 and 15:00. All participants fasted for at least 2 h before blood collection. Next, OC, IGF-1, and albumin levels were measured. Albumin was used as a nutritional marker. Blood analyses were performed at a laboratory within 24 h of extraction (Japan Clinical Laboratories, Inc., Kyoto, Japan). Total OC, IGF-1, and albumin levels were measured by electrochemiluminescence immunoassay, radioimmunoassay, and nephelometry, respectively.

### 2.5. Medical History

A self-completed medical history questionnaire regarding hypertension, diabetes mellitus, hyperlipidemia, osteoarthritis, and osteoporosis was used.

### 2.6. Assessment of Oral Dysfunction

The assessment of oral dysfunction was based on the subjective judgment of each participant in regard to each of the following items: “Do you have any difficulties eating tough foods compared to 6 months ago? (decline in masticatory function)”, “Have you choked on your tea or soup recently? (difficulty swallowing)”, and “Do you often experience having a dry mouth? (dry mouth)”. These items are in reference to a basic checklist developed for the purpose of evaluating the function of older people and predicting the risk of developing the need for nursing care [24].

### 2.7. Statistical Analysis

The study participants were categorized into a non-dysfunction and a dysfunction group for each questionnaire item about oral functions. IGF-1 levels between the masticatory non-dysfunction and dysfunction groups were compared using Welch’s *t*-test. The odds ratios (ORs) for oral dysfunction variables were calculated using univariate and multiple logistic regression analyses. Serum concentrations of physiological substances, GDS-15 scores, body composition parameters, and medical history were used as independent variables, and oral status was the dependent variable. The p values on multiple logistic regression analysis were corrected with the Bonferroni correction. The IGF-1 threshold concentration for discriminating a decline in masticatory function was evaluated using receiver operating characteristic curve (ROC) analysis. The ORs of IGF-1 levels for the masticatory dysfunction threshold score were calculated using logistic regression analyses. The correlation coefficients for serum substances and age, GDS-15 score, and body composition were calculated using Pearson correlation analysis. Statistical analysis was conducted using JMP 11 (SAS Institute, Cary, NC, USA). All statistical tests were two-tailed, and a significance level of 0.05 was used.

## 3. Results

### 3.1. Characteristics of the Study Participants

The age, serum physiological substance levels, body composition, medical history, and subjective oral function of the participants are shown in Table 1. In total, 23.02% of the participants had masticatory dysfunction, 28.06% had difficulty swallowing, and 25.18% had dry mouth (Table 1).

### 3.2. Odds Ratios for the Characteristics of Oral Dysfunction

**Figure 1:**
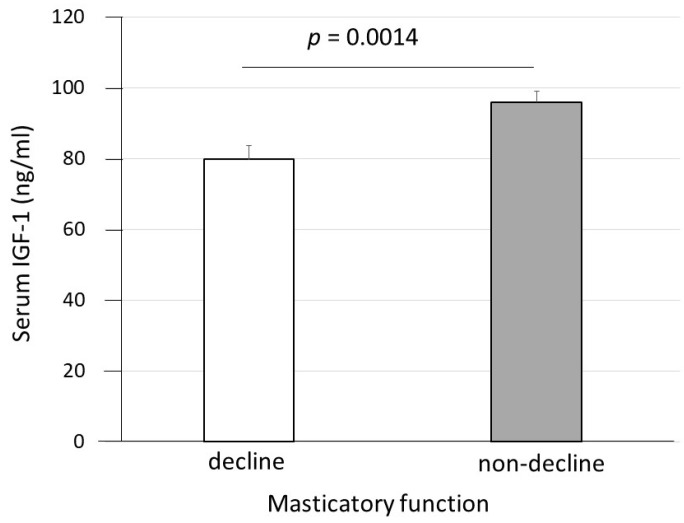
Comparison of IGF-1 levels between the masticatory dysfunction and non-dysfunction groups. Welch’s *t*-test was performed. IGF-1, insulin-like growth factor-1.

The results of the univariate and multiple logistic regression analyses regarding the ORs for all measurements, including age, serum physiological substance levels, physical function, body composition, and medical history of oral dysfunction, “decline in masticatory function”, “difficulty swallowing”, and “dry mouth” are shown in Table 2. The results of the univariate regression analysis showed that the IGF-1 level (OR = 0.976, 95% confidence interval [CI] = 0.958–0.994; *p* = 0.0308) and osteoporosis status (OR = 2.423, 95% CI = 1.060–5.537; *p* = 0.02086) were significantly associated with a “decline in masticatory function”. The results of multiple regression analysis also showed that the IGF-1 level (OR = 0.978, 95% CI = 0.960–0.996; *p* = 0.0308) was significantly associated with a “decline in masticatory function”. The results of the comparison of IGF-1 levels between the masticatory dysfunction and non-dysfunction groups are shown in Figure 1. IGF-1 levels were significantly higher in the non-dysfunction group (mean age ± SE, 96.103 ± 2.784 ng/mL) than in the dysfunction group (79.906 ± 5.090 ng/mL) (*p* = 0.0014) (Figure 1). In addition, age was significantly associated with “dry mouth” (OR = 1.082, 95% CI = 1.010–1.158; *p* = 0.0252). For “difficulty swallowing”, no significant associations were found (Table 2).

### 3.3. Threshold Score of IGF-1 Levels for a “Decline in Masticatory Function”

ROC analysis of the IGF-1 level showed a threshold of 108 ng/mL for discriminating a “decline in masticatory function” (area under the curve = 0.662, sensitivity = 96.88%, specificity = 30.84%, *p* < 0.0074) (Table 3).

### 3.4. Odds Ratios for a “Decline in Masticatory Function” According to IGF-1 Levels

Univariate logistic regression analysis showed that participants with an IGF-1 level ≤108 ng/mL had an OR of 4.311 for a “decline in masticatory function” (95% CI = 1.226–15.159; *p* = 0.0228) (Table 4).

### 3.5. Correlation between Serum Physiological Substance Levels and Variables

Table 5 shows the correlations between OC, IGF-1, albumin, and body composition parameters. IGF-1 levels were significantly negatively correlated with age (r = −0.219, *p* = 0.0097), and positively correlated with both SMI (r = 0.168, *p* = 0.0475) and BMD (r = 0.198, *p* = 0.0192) (Table 5).

## 4. Discussion

In this study, a negative correlation was found between serum IGF-1 levels and masticatory dysfunction. In particular, when the serum IGF-1 level was ≤108 ng/mL, masticatory dysfunction was found to have an OR of 4.3. The reference value for IGF-1 was 54–190 ng/mL [25], and this cutoff value was within the reference value range. No associations were found between IGF-1 levels and “difficulty swallowing” or “dry mouth” or between OC levels and oral dysfunction.

The masticatory system is a group of highly organized craniofacial structures, including bone (upper and lower jaws), teeth, joints, neurovascular elements, and four muscles: masseter, temporal, lateral pterygoid, and medial pterygoid muscles. IGF-1 has been suggested to be an important modulator of muscle mass and function not only during development, but also throughout life [26]. The serum IGF-1 concentration decreases with age [27]. In the present study, a positive correlation was found between blood IGF-1 levels and SMI (Table 5). Moriwaki et al. reported similar results [28]. It has also been reported that low serum IGF-1 levels increase the likelihood of developing sarcopenia [29]. In addition, many studies have reported that various exercises up-regulate IGF-1 expression [30,31].

On the other hand, the mandible is exposed to mechanical loads owing to mastication and occlusion. Regarding the relationship between bone and IGF-1, IGF-1 is the most abundant growth factor in the bone matrix and promotes osteoblast formation by exerting anabolic action on bone [32,33]. Blood IGF-1 has been reported to be positively correlated with calcaneal bone mass [28]. In the present study, a correlation was found between IGF-1 levels and BMD (Table 5). However, no relationship was observed between masticatory function and BMD (Table 2). This result suggests that the BMD of the calcaneus is related to serum IGF-1 levels but does not reflect masticatory function.

Several studies have been conducted to investigate the relationship between osteoporosis and reduced mastication [13,14]. The results of the present study also revealed a relationship between osteoporosis and decreased mastication (Table 2). From the above results, it was thought that the decrease in mastication was associated with a drastic decrease in BMD. Since decreased serum IGF-1 levels may be involved in decreased bone quality, combining serum IGF-1 with BMD may increase screening efficiency for risk assessments of diabetes-related osteoporosis [34]. Therefore, IGF-1 is an important modulator of bone metabolism and muscle mass and function in community-dwelling middle-aged and older adults [28].

Most IGF-1 is produced and secreted in the liver. However, as mentioned above, IGF-1 is also synthesized in bone and skeletal muscle. In particular, its expression has been reported to be up-regulated in these organs by mechanical loading [35]. In rodent studies, IGF-1 synthesis in the submandibular gland has also been reported, and this synthesis has been reported to decline with age [36]. On the other hand, increased mastication has been shown to induce IGF-1 in osteocytes of the jaw bone and suppress bone cell sclerostin (which has the effect of inhibiting bone formation), thereby enhancing osteoblast formation of tendon-derived cells [7]. These previous findings support our present results regarding the relationship between IGF-1 and masticatory function.

Based on the above results, in older people, a decrease in serum IGF-1 levels causes a decrease in locomotor organs, including bones and muscles, which in turn, reduces the amount and quality of jaw bone mass and occlusal muscles, resulting in masticatory dysfunction. Conversely, a deficiency of nutrients occurs as masticatory power decreases, which adversely affects the structure of the locomotor organs, thereby resulting in a decrease in serum IGF-1 levels. To the best of our knowledge, this is the first study to clarify the relationship between mastication and serum IGF-1 levels in humans. Since a decrease in masticatory power leads to frailty syndrome and cognitive decline in the aged, early detection of a decrease in masticatory power may suppress the progression of these pathological conditions. Blood IGF-1 concentration is therefore useful to judge the deterioration of masticatory function in aged persons.

The present study Had several limitations. First, The sample size (*n* = 139) was small. Therefore, additional research with a larger sample is needed. Second, the participants were all Japanese people; therefore, caution is needed when generalizing the results to other populations. Third, only three types of tooth function items were investigated this time. In the future, it will be necessary to investigate items such as tongue pressure and objective oral conditions (e.g., number of teeth, periodontal status, occlusal support, and quantification of saliva).

## 5. Conclusions

The results of this study suggest a potential relationship between lower levels of serum IGF-1 and masticatory dysfunction in community-dwelling older people, regardless of age, depression, or body composition. Therefore, the serum IGF-1 levels might be a useful marker for masticatory function in older people. However, validation by quantitative measurement of oral function is important.

## Figures and Tables

**Table 1:** Characteristics of the study participants.

	Mean/n	SD/%
Participants (male %)	139	18.00%
Age (years)	74.16	5.70
OC (ng/mL)	17.00	6.64
IGF-1 (ng/mL)	92.37	29.50
Albumin (g/dL)	4.30	0.25
GDS-15 (points) (*n* = 129)	3.10	2.46
BMI (kg/m^2^)	22.98	3.19
SMI (kg/m^2^)	5.95	0.94
BFP (%)	30.92	7.41
BMD (%YAM)	86.65	11.10
Hypertension	63	45.32%
Diabetes mellitus	10	7.19%
Hyperlipidemia	41	29.50%
Osteoarthritis	25	17.99%
Osteoporosis	40	28.78%
Masticatory dysfunction	32	23.02%
Difficulty swallowing	39	28.06%
Dry mouth	35	25.18%

Notes. Values are presented as mean (SD) or prevalence (%) values. SD, standard deviation; OC, osteocalcin; IGF-1, insulin-like growth factor-1; GDS-15, Geriatric. Depression Scale; BMI, body mass index; SMI, skeletal muscle mass index; BFP, body fat percentage; BMD, bone mineral density; YAM; young adult mean.

**Table 2:** Odds ratios of participants’ characteristics for oral dysfunction.

	Masticatory Dysfunction						Difficulty Swallowing			Dry Mouth		
	Univariate			Multiple			Univariate			Univariate		
	OR	95% CI	*p*	OR	95% CI	*p* *	OR	95% CI	*p*	OR	95% CI	*p*
Age	1.060	0.989–1.136	0.0995				1.049	0.983–1.120	0.1483	1.082	1.010–1.159	0.0252
Sex (male)	1.384	0.520–3.686	0.5150				0.996	0.380–2.613	0.9940	1.516	0.590–3.900	0.3878
OC	0.990	0.932–1.052	0.7533				0.978	0.923–1.035	0.4400	1.013	0.957–1.073	0.6524
IGF-1	0.976	0.958–0.994	0.0074	0.978	0.960–0.996	0.0308	1.000	0.991–1.016	0.5325	0.996	0.982–1.009	0.5281
Albumin	0.368	0.077–1.766	0.2114				3.271	0.696–15.37	0.0770	0.343	0.074–1.586	0.1709
GDS-15	0.998	0.961–1.036	0.9159				1.008	0.973–1.044	0.6548	1.008	0.972–1.045	0.6589
BMI	0.992	0.876–1.124	0.8990				1.009	0.898–1.133	0.8807	0.987	0.875–1.114	0.8372
SMI	0.800	0.097–6.775	0.8457				0.731	0.441–1.212	0.2240	0.878	0.516–1.49	0.6326
BFP	1.012	0.958–1.069	0.6646				1.017	0.966–1.071	0.5172	1.014	0.961–1.069	0.6146
BMD	0.988	0.951–1.025	0.5161				1.007	0.975–1.041	0.6605	0.978	0.941–1.016	0.2793
Hypertension	0.659	0.929–1.481	0.3126				0.794	0.371–1.659	0.5254	0.72	0.334–1.553	0.4025
Diabetes mellitus	0.825	0.166–4.096	0.8140				0.622	0.126–3.066	0.5593	0.727	0.147–3.599	0.6963
Hyperlipidemia	1.617	0.702–3.712	0.2601				1.779	0.812–3.899	0.1503	1.349	0.595–3.061	0.4734
Osteoarthritis	1.384	0.520–3.686	0.5150				1.575	0.630–3.940	0.3315	1.516	0.590–3.900	0.3878
Osteoporosis	2.423	1.060–5.537	0.0358	2.033	0.864–4.784	0.2086	1.143	0.509–2.564	0.7460	0.987	0.423–2.302	0.9752

Notes. Univariate and multiple logistic regression analyses were performed. * corrected with Bonferonni. OR, odds ratio; CI, confidence interval; OC, osteocalcin; IGF-1, insulin-like growth factor-1; GDS-15, Geriatric Depression Scale; BMI, body mass index; SMI, skeletal muscle mass index; BFP, body fat percentage; BMD, bone mineral density.

**Table 3:** Threshold of the IGF-1 level for a “decline in masticatory function”.

IGF-1 (ng/mL)	AUC	Sensitivity	Specificity	*p*
108	0.662	96.88	30.84	0.0074

Notes. Receiver operating characteristic curve (ROC) analysis was performed. AUC, area under the ROC curve; IGF-1, insulin-like growth factor-1.

**Table 4:** Odds ratios of IGF-1 (≤108 ng/mL) for a “decline in masticatory function”.

	OR	95% CI	*p*
IGF-1 (≤108 ng/mL)	4.311	1.226–15.159	0.0228

Notes. Logistic regression analyses were performed. OR, odds ratio; IGF-1, insulin-like growth factor-1; CI, confidence interval.

**Table 5:** Correlation coefficients for serum substances and age, GDS-15 score, and body composition parameters.

	Age	GDS-15	BMI	SMI	BFP	BMD
OC	−0.047 (0.5847)	0.047 (0.5932)	−0.051 (0.5496)	−0.104 (0.2228)	−0.001 (0.9894)	−0.126 (0.1405)
IGF-1	**−0.219** (0.0097)	−0.055 (0.5332)	0.166 (0.0507)	**0.168** (0.0475)	0.062 (0.4662)	**0.198** (0.0192)
Albumin	−0.150 (0.0787)	0.069 (0.4358)	−0.015 (0.8635)	0.040 (0.6367)	0.014 (0.8652)	0.133 (0.1188)

Notes. Values are Pearson’s correlation coefficients (*p* value). Significant *p* values are shown in bold. OC, osteocalcin; IGF-1, insulin-like growth factor-1; GDS-15, Geriatric Depression Scale; BMI, body mass index; SMI, skeletal muscle mass index; BFP, body fat percentage; BMD, bone mineral density.

## Data Availability

The database used and analyzed during the present study is available from the corresponding author on reasonable request.
